# Predisposition to Cervical Atypia in Systemic Lupus Erythematosus: A Clinical and Cytopathological Study

**DOI:** 10.1155/2015/751853

**Published:** 2015-07-09

**Authors:** Hend Hilal Al-Sherbeni, Ahmed Mohamed Fahmy, Nadine Sherif

**Affiliations:** ^1^Rheumatology and Rehabilitation Department, Faculty of Medicine, Cairo University, Cairo, Egypt; ^2^Pathology Department, National Cancer Institute (NCI), Cairo University, Cairo, Egypt; ^3^Gynecology and Obstetrics Department, Faculty of Medicine, Cairo University, Cairo, Egypt

## Abstract

*Introduction*. Systemic lupus erythematosus (SLE) is a complex disease with variable presentations, course, and prognosis. The female genital tract may be a potential target organ in SLE since cervical inflammation may be associated with disease activity. An increase in cervical dysplasia, a precursor of cervical cancer, has been reported in females with SLE. *Aim of the Work*. This work aimed to study the prevalence of abnormal cervicovaginal smears in patients with systemic lupus erythematosus (SLE) and to correlate abnormal smear findings with exposure to infection with human papilloma virus (HPV) in SLE patients. *Patients and Methods*. Thirty-two patients with SLE, fulfilling the 1997 revised criteria for the classification of SLE, were included in this study. They were subjected to full history taking, clinical examination, laboratory investigations, and cervicovaginal smearing. Twenty healthy subjects not known to suffer from any rheumatological disease were used as controls, and they were subjected to cervicovaginal smearing. *Results*. Four out of 32 SLE patients showed abnormal Pap smears (12.5%) compared to none showing any cervical changes in the control group (0%). Among these 4 patients, 3 were having ASCU and one was having LSIL (HPV). *Conclusion*. Cervicovaginal smearing is an easy, economic, safe, repeatable, and noninvasive technique for screening and early detection of cervical neoplastic lesions in SLE.

## 1. Introduction

Systemic lupus erythematosus (SLE) is an autoimmune disease which is characterized by immune system disturbance. Physicians have been forced to deal with long term complications of SLE disease itself or complications due to its treatment in spite of improved survival rates [1].

Cervical cancer is the second most common cause of death worldwide (after breast cancer) [2]. Cervical cancer constitutes also 25% of female cancers in developing countries and is considered the most common cancer [3]. The association between human papilloma virus (HPV) and cervical squamous cell cancer is higher than the association between smoking and lung cancer [4]. HPV has been implicated for 99.7% of cervical squamous cell cancer worldwide [5].

As HPV cannot be cultured in the laboratory from clinical specimens, the primary diagnostic tools have been cytology and histology in the form of Pap smear [6].

The cervicovaginal smear or Papanicolaou-stained (Pap) smear is named after the pathologist George Papanicolaou, who introduced the method in 1949 before the cause of cervical cancer is known [7]. Since its introduction, the Pap smear has helped in reducing the cervical cancer incidence and mortality rates by half to two-thirds [8]. Pap smear is a screening tool that detects the changes in cells of the transformation zone of the cervix. Mostly, these changes are caused by HPV.

An important complication of SLE is increased incidence of malignancy including cervical cancers [9]. Autoimmune system dysfunction, disease activity, recurrent infections, and immunosuppressive medication exposure may constitute explanations which suggest increased incidence of malignancy in SLE [10].

Women having SLE have a higher rate of infection with HPV than others. And so lupus patients have a higher incidence of abnormal Pap smears than others not having lupus. This rate is significantly higher among women in the first five years following their diagnosis than for those who had lupus for more than 10 years [11].

## 2. Patients and Methods

Thirty-three cases, proven to be SLE patients, fulfilling the ACR criteria for classification of SLE [12], and attending the Inpatient Unit of Rheumatology and Rehabilitation Department, Faculty of Medicine, Cairo University hospitals, in the period between January 2011 and September 2011, were included in this study.

Inclusion criteria included the following:

- SLE female patients,
- females in the childbearing period,
- females not pregnant at the time of the Pap smear intake,
- females consenting to be included in the study.

Exclusion criteria included the following:

- patients with bleeding,
- pregnant ladies,
- patients refusing examination or participation in the study,
- virgin or menopausal ladies.

Patients included in the study were subjected to full history taking, general examination, local and bimanual examination, speculum examination with Pap smear intake (see Section 2.1), and full laboratory workup. Informed consent was obtained from all patients after full explanation of the aim and procedures of the study; data were collected after informing the patients about the purpose of the study; the patients had the right to withdraw from the study without giving any reasons. Privacy and confidentiality of the obtained data was insured for all participants. The ethical committee of Kasr Al Ainy hospitals approved this study before starting.

### 2.1. Technique

Patients were examined in the examination room, in the lithotomy position, using bimanual examination to determine the size of the uterus, its direction, and any feeling of abnormalities in the uterus or cervix; then exposure of the cervix by Cusco's speculum was done, followed by cleaning with isotonic saline solution and the intake of the Pap smear.

Pap smear test is based on cytological examination of cells shed from the ectocervix. In this study, it was performed using a cytobrush to wipe cells from the ectocervical canal and an Ayre spatula to wipe cells from the surface of the ectocervix. Cells obtained were spread on 4 slides (2 normal slides and another 2 positively charged slides). These cells were then fixed by ethyl alcohol and stained with special stains.

Pap smear is an office procedure, with an accuracy rate > 80%; however, it carries a small percentage of both false positive and false negative results (15–25%) [13, 14]. Smears were stained by hematoxylin and eosin (H&E) stain.

### 2.2. Immunostaining

Smears were immunostained using labeled streptavidin biotin (LSAB Kit, Dako) according to the provided manufacturers' manual. The smear slides were fixed overnight in absolute alcohol. After blocking endogenous peroxidase, human papilloma virus (HBV) ab-3 monoclonal antibody, ready to use (Thermo scientific, USA), was applied and incubated for 30 minutes, followed by secondary biotinylated antibody for 30 minutes. 3,3′-Diaminobenzidine tetrahydrochloride (DAB) solution was used as the final chromogen.

### 2.3. Immunostaining Analysis

Immunostaining results were considered positive nuclear (+) or negative nuclear (−) staining with a positive control used with each run provided by the manufacturer. Negative controls for nonspecific binding, incubated with secondary antibodies only, were processed and revealed no signals.

## 3. Results

This study included 32 patients with SLE; all were females. Their ages ranged from 21 to 50 years with a mean of 31.06 ± 7.170 years. Disease duration ranged from 1 year to 16 years with a mean of 6.13 ± 4.139 years. A group of 20 age matched females served as control. Their ages ranged from 20 to 45 years with a mean of 30.2 ± 7.5 years.

The clinical and laboratory features of SLE patients reported in this study were obtained at time of Pap smears (Tables 1, 2, and 3).

15/32 (46.9%) patients showed positive changes in renal biopsy and they were as follows: one patient showed grade I renal affection, two patients showed grade II, one patient showed grade I-II, one patient showed grade II–V, six patients showed grade III, and one patient showed grade III-IV, while three patients had grade IV renal affection.

21/32 (65.6%) patients were receiving azathioprine (Imuran) with a dose ranging from 50 to 150 mg with a mean of 100 ± 15.811. 29/32 (90.6%) patients were receiving hydroxychloroquine (Hydroquine) with a dose ranging from 200 to 400 mg with a mean of 379.31 ± 61.987. 17/32 (53.1%) received pulse cyclophosphamide therapy. All patients (100%) were receiving steroids with a dose ranging from 10 to 60 mg with a mean of 25.23 ± 10.632.

SLEDAI activity score was performed to SLE patients at study time and was as follows:

- 20 out of 32 patients (62.5%) had mild disease activity,
- 10 out of 32 patients (31.25%) had moderate disease activity,
- 2 out of 32 patients (6.25%) had severe activity of SLE.

### 3.1. Results of Cervicovaginal Smear Examination

**Figure 1:**
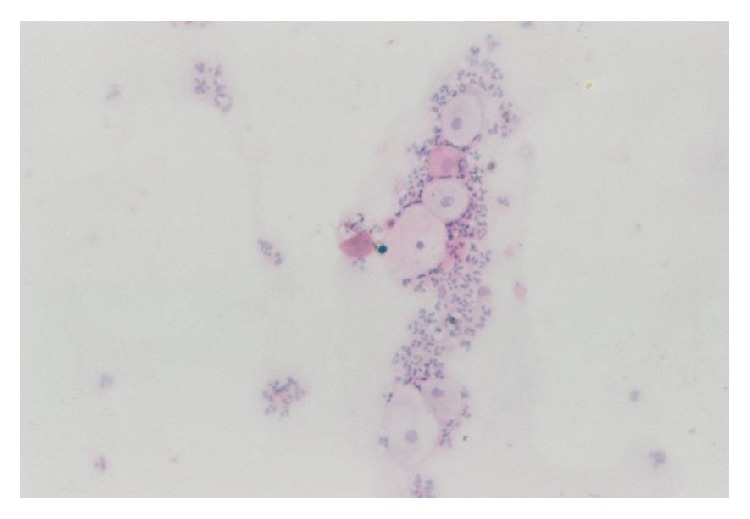
Superficial, intermediate, and parabasal epithelial cells (H&E, ×400).

**Figure 2:**
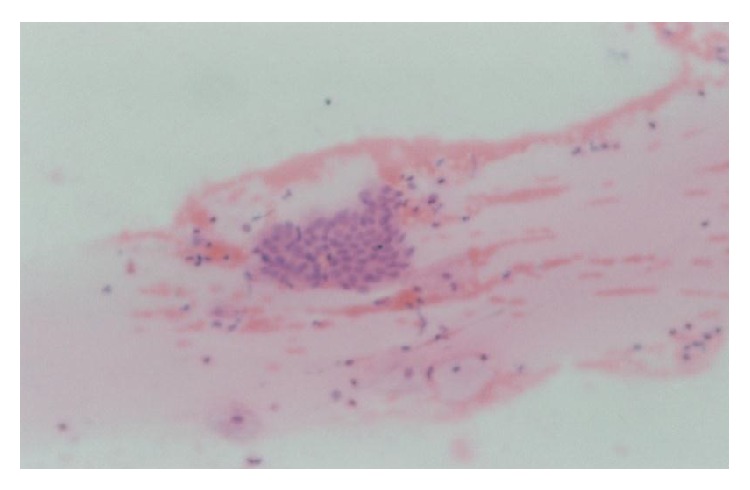
Groups of endocervical cells in cervicovaginal smear (HX, ×100).

**Figure 3:**
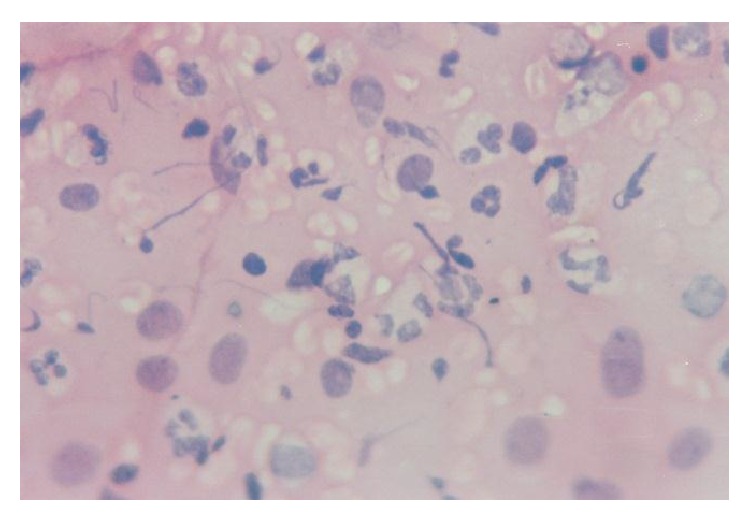
Atypical squamous cells of undetermined origin (ASCUS) (H&E, ×400).

**Figure 4:**
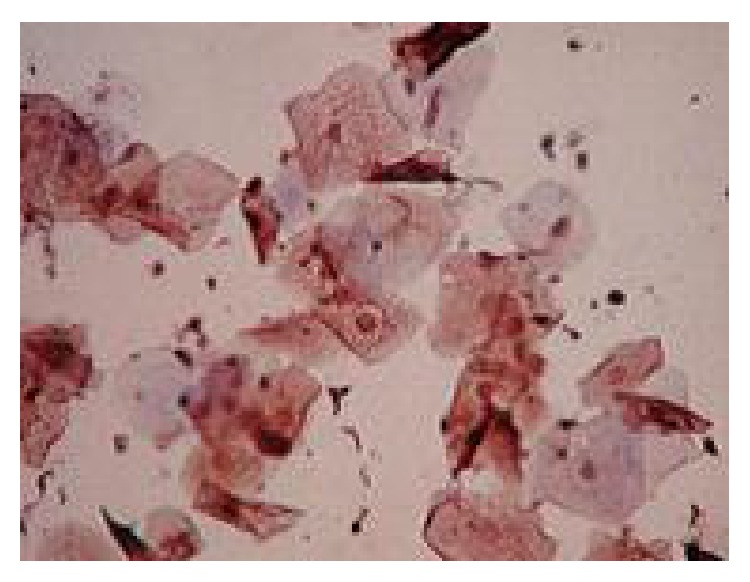
A case with HPV type 16 infected cells showing koilocytic changes (CIN I) (×400).

All cases showed adequate cellular smears with a mixture of superficial, intermediate, and parabasal epithelial cells (Figure 1). Adequacy of smears was determined by the presence of groups of endocervical cells (Figure 2). Neutrophilic infiltration ranged from mild to marked infiltration with no evidence of specific genital infection in any of the cases. Three cases showed ASCUS cells (Figure 3). One case showed low grade squamous intraepithelial lesion (low grade dysplasia, low grade CIN) with koilocytic changes. This was confirmed by positive nuclear immunostaining for HPV type 16 (Figure 4).

### 3.2. Cyclophosphamide Exposure

Despite obvious tendency of SLE patients to develop abnormal cervicovaginal smears, especially after CYC exposure, no statistical significant correlation was found between systemic lupus erythematosus and abnormal cervicovaginal smears nor between CYC and abnormal cervicovaginal smears which could be explained by small number of patients in our study.

## 4. Discussion

In this study, we want to find out whether lupus patients are at increased risk of abnormal Pap smears and whether they are also at increased risk of HPV infections specially the oncogenic subtypes (16, 18). Also we want to highlight the effect of different medications (cyclophosphamide, hydroxychloroquine, and steroids) on Pap smear and cervical changes.

Our study showed that there are 4 out of 32 SLE patients, having abnormal Pap smear (12.5%); this result is comparable with Esmaeili and Ghahremanzadeh who found that the frequency of abnormal Pap smear testing was higher in patients with SLE (8.1%) compared to control group [15].

The present study showed only one case of HPV type 16, having abnormal Pap smear in the form of LSIL (low grade intraepithelial lesion) among the whole 32 cases studied. This cannot be depending on regarding confirming the increased incidence of HPV infection, specially the oncogenic type with SLE disease; however, the study done by Nath et al. in 2007 showed that UK women with a recent SLE diagnosis had disturbingly elevated levels of HPV infections (particularly with HPV 16), abnormal cervical cytology, and SIL. Larger sample size in our population may have confirmed the results of Nath study [16]. The same study showed also the higher incidence of abnormal Pap smear in women with SLE, especially in the first 5 years of diagnosis of SLE; in our results, we had 4 patients with abnormal Pap smear in the form of ASCUS and LSIL.

Previous studies showed the effect of different medications used to treat SLE patients on the cervical changes and hence the Pap smear results. Bateman et al. [17] showed that there was a significant decrease in time to dysplasia in patients given IV-C (intravenous cyclophosphamide), with previous dysplasia compared to those without. These preliminary data suggest that IV-C causes an increased number of abnormal Pap smear in SLE patients, particularly those with previous dysplasia. In our study, 17 out of 32 patients were receiving IV-C, and we have 4 patients with cervical dysplasia; one of them was receiving IV-C. So due to these small numbers, we could not state a statistical correlation between IV-C and cervical changes.

Other studies, as Nath et al. [16], stated, similar to us, that it was not possible for them to interpret whether their patients treated with hydroxychloroquine and having cervical changes are having these changes due to the medications used or due to the SLE disease itself, specially that usually the patient is receiving a combination of more than one drug.

Regarding the limitations in our study, the main issue is the small number of the studied population; also they were all receiving medications (21 were receiving azathioprine, 29 were receiving hydroxychloroquine, 17 were receiving cyclophosphamide, and 32 were receiving steroids), so we can never confirm whether the cervical changes were due to the disease SLE itself or due to the medications taken.

Despite its limitation and its small sample size, this study provides further evidence that women having SLE are at increased risk of developing cervical changes which may be HPV of oncogenic subtype compared to their equivalent (4 out of 32 patients with SLE developed cervical changes, compared to none out of 20 control). And as Pap smear is a simple, safe, repeatable procedure, it is recommended regularly to all SLE patients, especially in the first 5 years of diagnosis.

## Figures and Tables

**Table 1:** Showing percentage of each clinical manifestations of SLE patients.

Clinical feature	Number of patients	%
Constitutional manifestations		
(Fever and fatigue)	32	100%
Mucocutaneous manifestations		
Malar rash	30	93.8%
Oral ulcers	22	68.8%
Alopecia	6	18.75%
Musculoskeletal manifestations		
Arthritis	31	96.9%
Vascular manifestations		
Raynaud's phenomenon	5	15.625%
Systemic hypertension	5	15.625%
Pulmonary manifestations		
Pleurisy	20	62.5%
Pleural effusion	1	3.125%
Cardiac manifestations		
Pericarditis	1	3.125%
Pericardial effusion	0	0%
Renal involvement		
Proteinuria	20	62.5%
Urinary casts	8	25%
Neurological manifestations		
Headache	12	37.5%
Seizure	1	3.125%
Stroke	1	3.125%
Psychosis	1	3.125%
Hematological manifestations		
Anemia	9	28.1%

**Table 2:** Showing laboratory data of SLE patients.

Lab	Minimum	Maximum	Mean	±SD
HB	6 gm/dL	14.2	10.309	1.9734
TLC	1.9 × 10^3^/mm^3^	21.3 × 10^3^	7.497 × 10^3^	4.4902 × 10^3^
PLT	55 × 10^3^/mm^3^	442 × 10^3^	260.59 × 10^3^	93.832 × 10^3^
ALT	7 IU/mL	83 IU/mL	21.47 IU/mL	15.110 IU/mL
Creatinine	0.4	2.98	0.9356	0.61415

**Table 3:** Showing autoimmune profile and complement.

	Number of patients	Percentage
Positive ANA	27	84.4%
Positive anti-DNA	14	43.8%
Consumed C3	5	15.6%
